# Supplementary material for: polyBERT: a chemical language model to enable fully machine-driven ultrafast polymer informatics
Source: Nat Commun. 2023 Jul 11;14:4099. doi: 10.1038/s41467-023-39868-6 (PMC10336012; doi:10.1038/s41467-023-39868-6)
Supplement: Supplementary file 1 — Supplementary Information [file 41467_2023_39868_MOESM1_ESM.pdf]

Supplementary Information

polyBERT: A chemical language model to enable fully machine-driven ultrafast polymer informatics

Christopher Kuenneth[1,2] and Rampi Ramprasad[*,1]

[1] *School of Materials Science and Engineering, Georgia Institute of Technology, Atlanta, Georgia 30332, USA*

[2] *Faculty of Engineering Science, University of Bayreuth, 95447 Bayreuth, Germany*

* E-mail: rampi.ramprasad@mse.gatech.edu

Supplementary Discussion

During the development of polyBERT, we evaluated the BERT,[1] RoBERTa,[2] DistilBERT,[3] and DeBERTa[4] models as the foundations of polyBERT. We trained property predictors for the homopolymer part of the data set described in Table 1 of the main manuscript. Comparing the coefficients of determination (R^2s), we found DeBERTa perform best, followed by RoBERTa, DistilBERT, and BERT. This result was not surprising and matches performance tests carried out in the original publication of DeBERTa.[4]

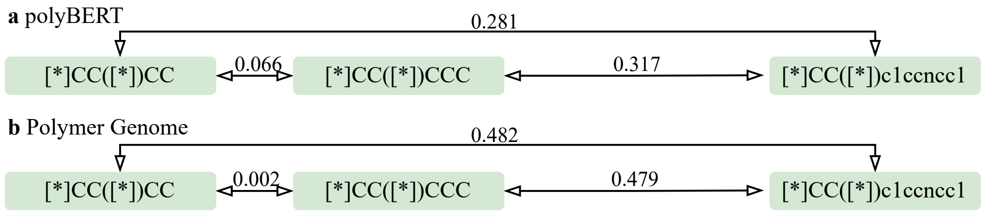

Supplementary Figure S1: Cosine distances between polymers fingerprint of Figure 2 in the main manuscript. The PSMILES strings `[*]CC([*])CC`, `[*]CC([*])CCC`, and `[*]CC([*])c1ccncc1` denote poly(but-1-ene), poly(pent-1-ene), and poly(4-vinylpyridine), respectively.

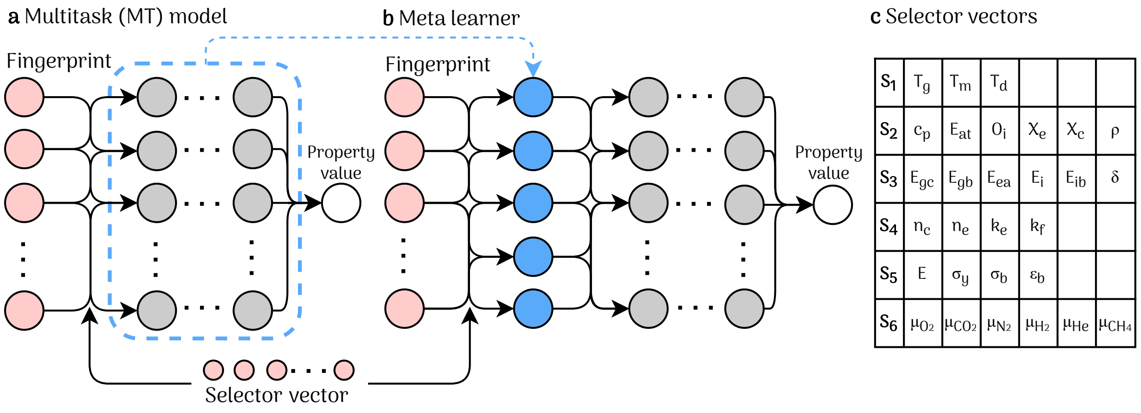

Supplementary Figure S2: Learning workflow. **a** Multitask model. We use five-fold cross-validation to train five multitask models. The cross-validation process ensures that each data point has once been in the validation data set and allows us to report the performance errors as averaged root-mean-square errors (RMSEs) and coefficients of determination (R^2s) of the five validation data sets. **b** Meta learner. The meta learner includes the five multitask models (blue nodes) with fixed weights. The outputs of these five multitask models are the inputs to a feed-forward network (grey nodes). Six meta learners with the selector vectors $\mathbf{S}_1$ to $\mathbf{S}_6$ operate as production predictors. **c** Selector vectors. $\mathbf{S}_i$ are binary vectors that select the output property. It is 1 for the selected property and 0 everywhere else, except for k_f where the $\log_{10}$ frequency is used instead of the 1. For instance, the selector vector of the thermal properties predictor ($\mathbf{S}_1$) has three components and encodes T_g as $(100)^T$, T_m as $(010)^T$, and T_d as $(001)^T$. T_g, T_m, and T_d of $\mathbf{S}_1$ stand for glass transition temperature, melting temperature, and degradation temperature. c_p, E_at, O_i, X_c, X_e, and ρ of $\mathbf{S}_2$ stand for heat capacity, atomization energy, limiting oxygen index, crystallization tendency (DFT), crystallization tendency (exp.), and density. E_gc, E_gb, E_ea, E_i, E_ib, and δ of $\mathbf{S}_3$ stand for band gap (chain), band gap (bulk), electron affinity, ionization energy, electronic injection barrier, and cohesive energy density. n_c, n_e, k_c, and k_f of $\mathbf{S}_4$ stand for refractive index (DFT), refractive index (exp.), dielectric constant (DFT), and dielectric constant at freq. **f**. E, σ_y, σ_b, and ϵ_b of $\mathbf{S}_5$ stand for young's modulus, tensile strength at yield, tensile strength at break, and elongation at break. μ_{O_2}, μ_{CO_2}, μ_{N_2}, μ_{H_2}, μ_He, and μ_{CH_4} of $\mathbf{S}_6$ stand for O_2 gas permeability, CO_2 gas permeability, N_2 gas permeability, H_2 gas permeability, He gas permeability, and CH_4 gas permeability.

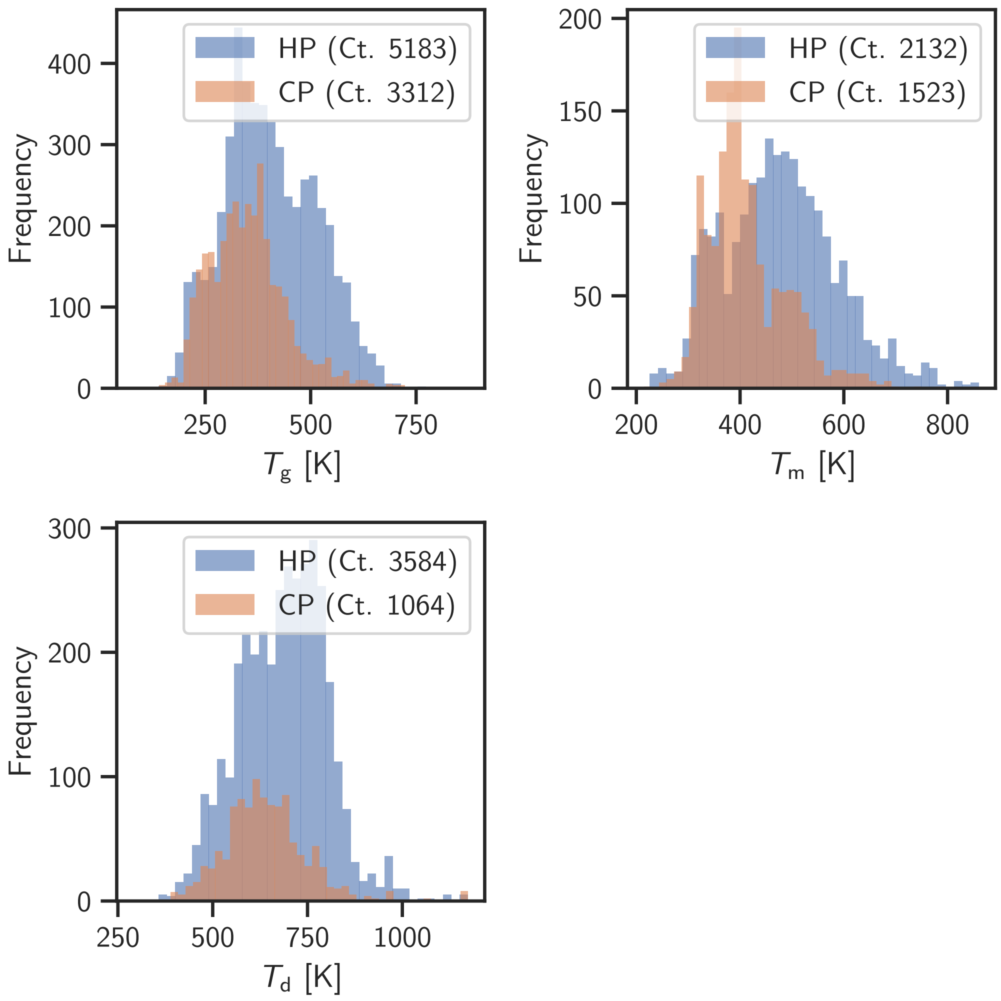

Supplementary Figure S3: Histograms of the data points of the thermal properties. HP and CP stand for homopolymer and copolymer, respectively. T_g, T_m, and T_d stand for glass transition temperature, melting temperature, and degradation temperature.

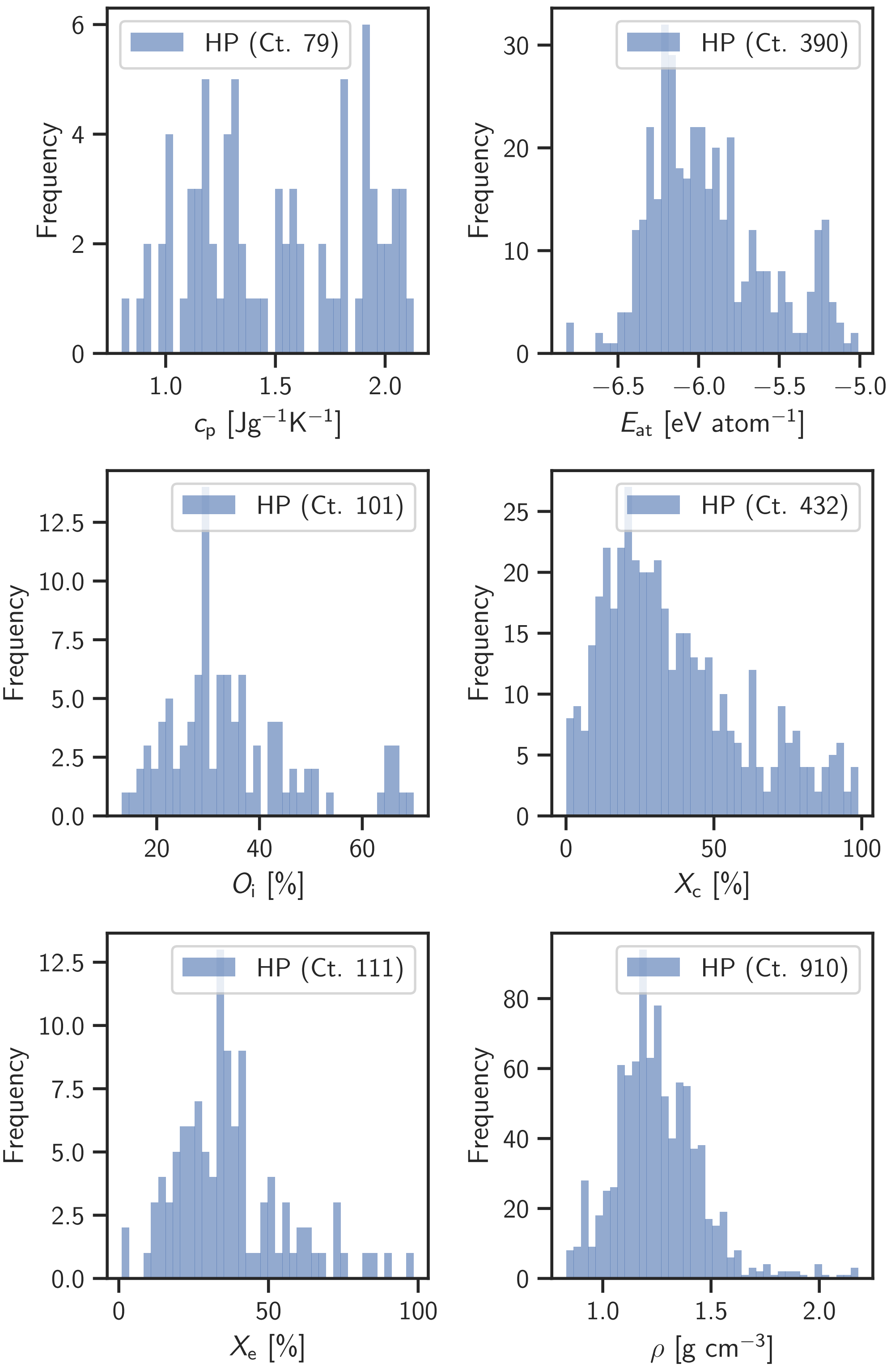

Supplementary Figure S4: Histograms of the data points of the thermodynamic & physical properties. HP stands for homopolymer. c_p, E_{at}, O_i, X_c, X_e, and ρ stand for heat capacity, atomization energy, limiting oxygen index, crystallization tendency (DFT), crystallization tendency (exp.), and density.

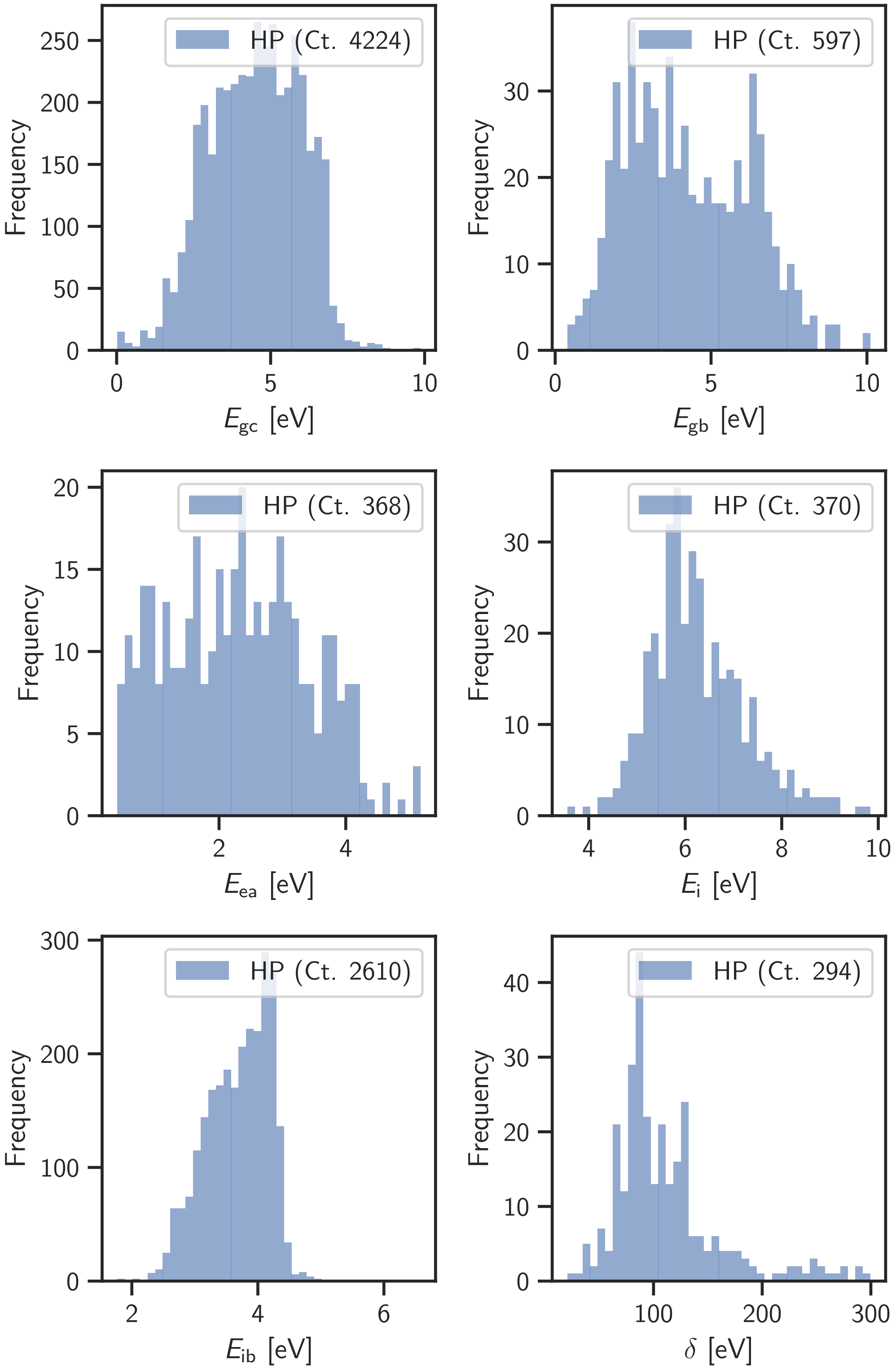

Supplementary Figure S5: Histograms of the data points of the electronic properties. HP stands for homopolymer. E_{gc}, E_{gb}, E_{ea}, E_{i}, E_{ib}, and δ stand for band gap (chain), band gap (bulk), electron affinity, ionization energy, electronic injection barrier, and cohesive energy density.

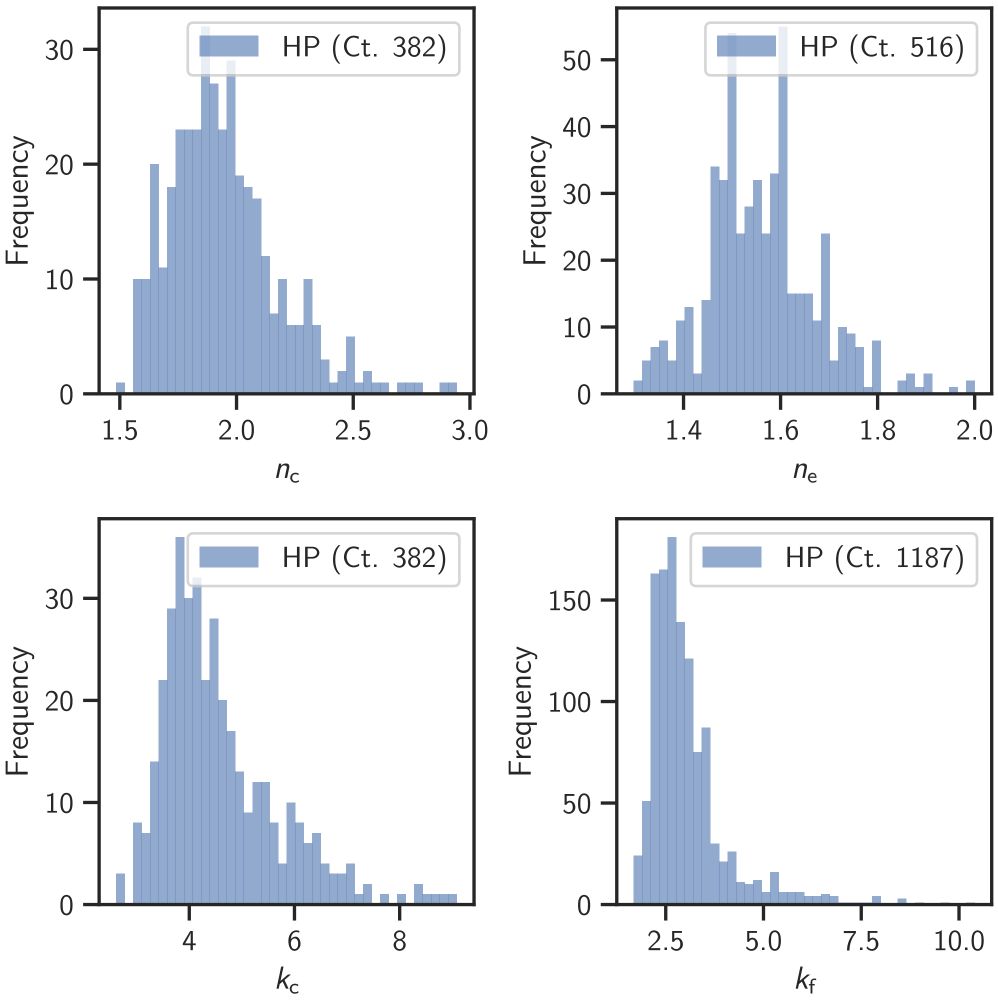

Supplementary Figure S6: Histograms of the data points of the optical & dielectric properties. HP stands for homopolymer. n_c, n_e, k_c, and k_f stand for refractive index (DFT), refractive index (exp.), dielectric constant (DFT), and dielectric constant at freq. **f**.

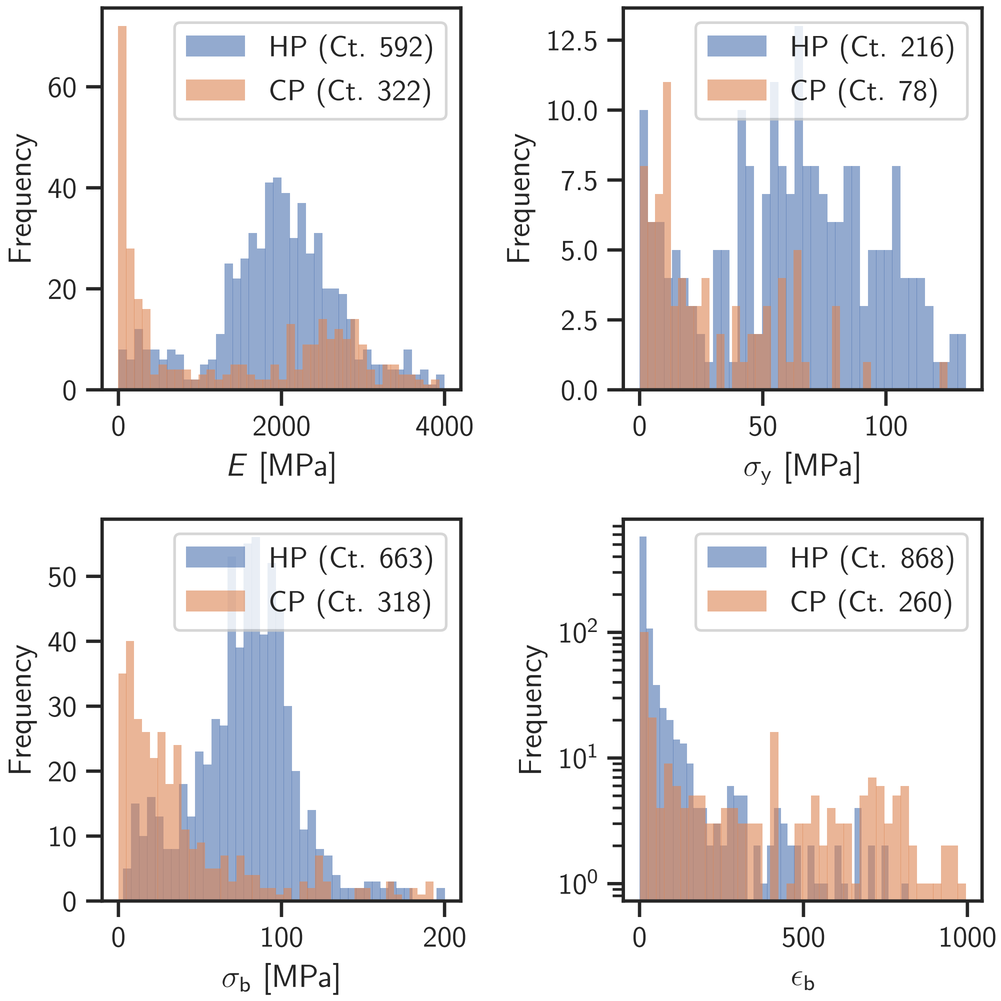

Supplementary Figure S7: Histograms of the data points of the mechanical properties. HP and CP stand for homopolymer and copolymer, respectively. E, σ_{y}, σ_{b}, and ϵ_{b} stand for young's modulus, tensile strength at yield, tensile strength at break, and elongation at break.

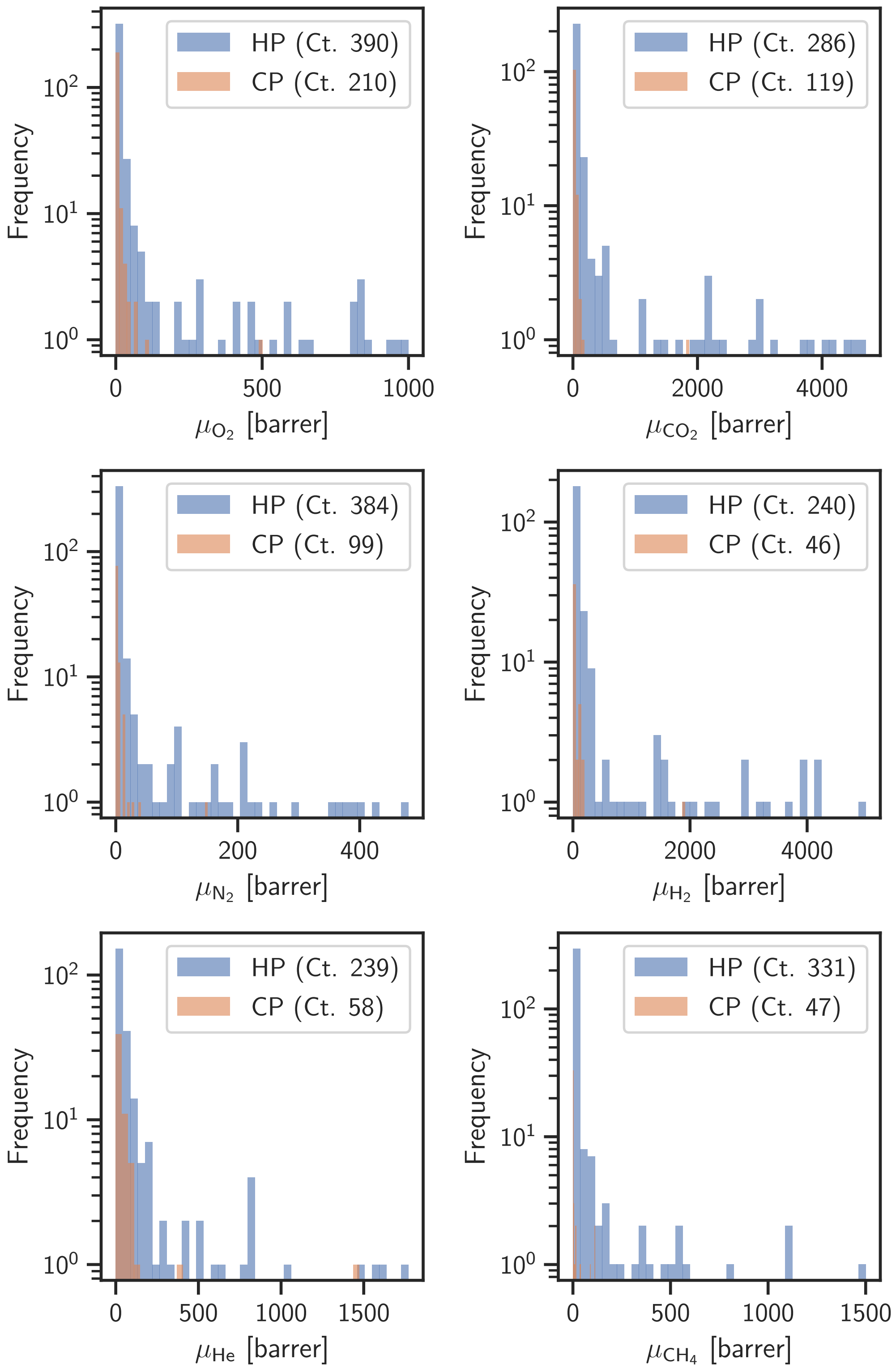

Supplementary Figure S8: Histograms of the data points of the permeability properties. HP and CP stand for homopolymer and copolymer, respectively. μ_{O_2}, μ_{CO_2}, μ_{N_2}, μ_{H_2}, μ_{He}, and μ_{CH_4} stand for O_2 gas permeability, CO_2 gas permeability, N_2 gas permeability, H_2 gas permeability, He gas permeability, and CH_4 gas permeability.

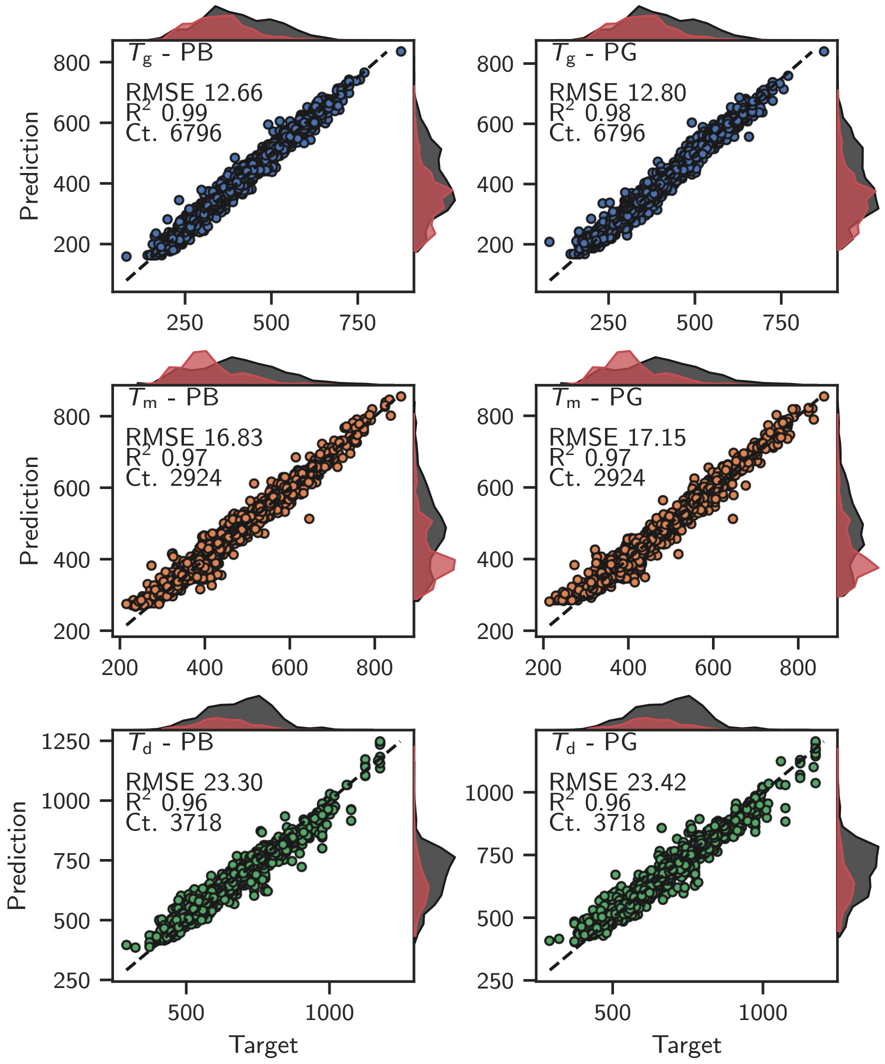

Supplementary Figure S9: Meta learner parity plots of the thermal properties' test data sets for polyBERT (PB) and Polymer Genome (PG) fingerprints. Data point frequencies of homopolymers and copolymers are indicated in black and red in the margins of the plots. RMSE, R^2, and Ct. denote the root-mean-square error, coefficient of determination, and number of data points. T_g, T_m, and T_d stand for glass transition temperature, melting temperature, and degradation temperature.

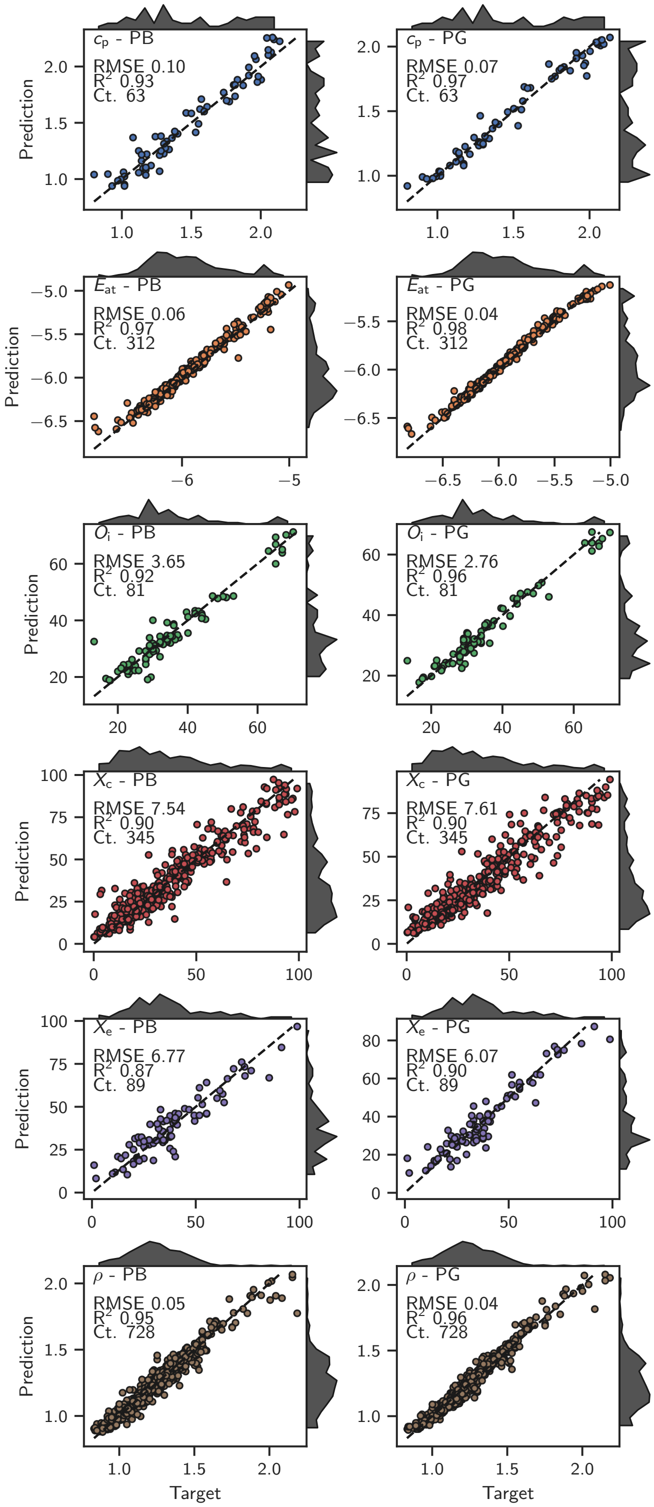

Supplementary Figure S10: Meta learner parity plots of the thermodynamic & physical properties' test data sets for polyBERT (PB) and Polymer Genome (PG) fingerprints. Data point frequencies of homopolymers are indicated in black in the margins of the plots. RMSE, R^2, and Ct. denote the root-mean-square error, coefficient of determination, and number of data points. c_p, E_{at}, O_i, X_c, X_e, and ρ stand for heat capacity, atomization energy, limiting oxygen index, crystallization tendency (DFT), crystallization tendency (exp.), and density.

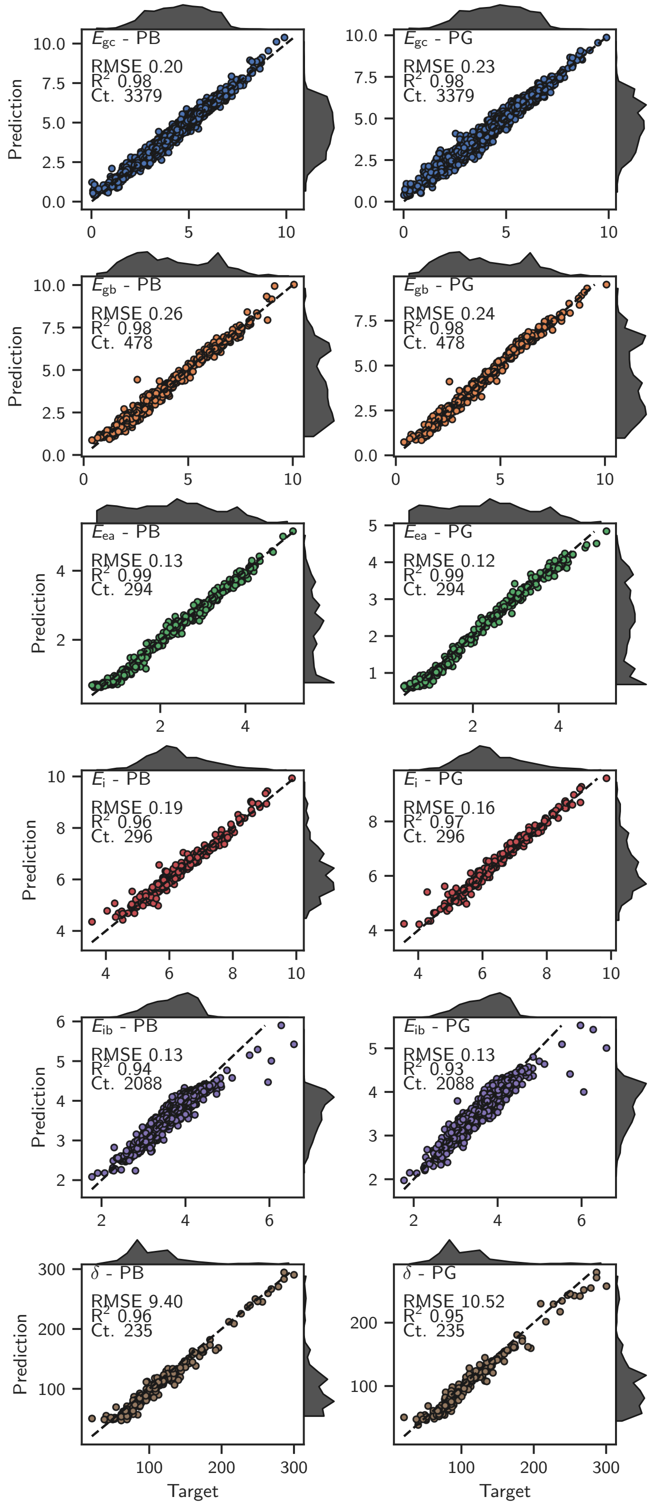

Supplementary Figure S11: Meta learner parity plots of the electronic properties' test data sets for polyBERT (PB) and Polymer Genome (PG) fingerprints. Data point frequencies of homopolymers are indicated in black in the margins of the plots. RMSE, R^2, and Ct. denote the root-mean-square error, coefficient of determination, and number of data points. E_{gc}, E_{gb}, E_{ea}, E_{i}, E_{ib}, and δ stand for band gap (chain), band gap (bulk), electron affinity, ionization energy, electronic injection barrier, and cohesive energy density.

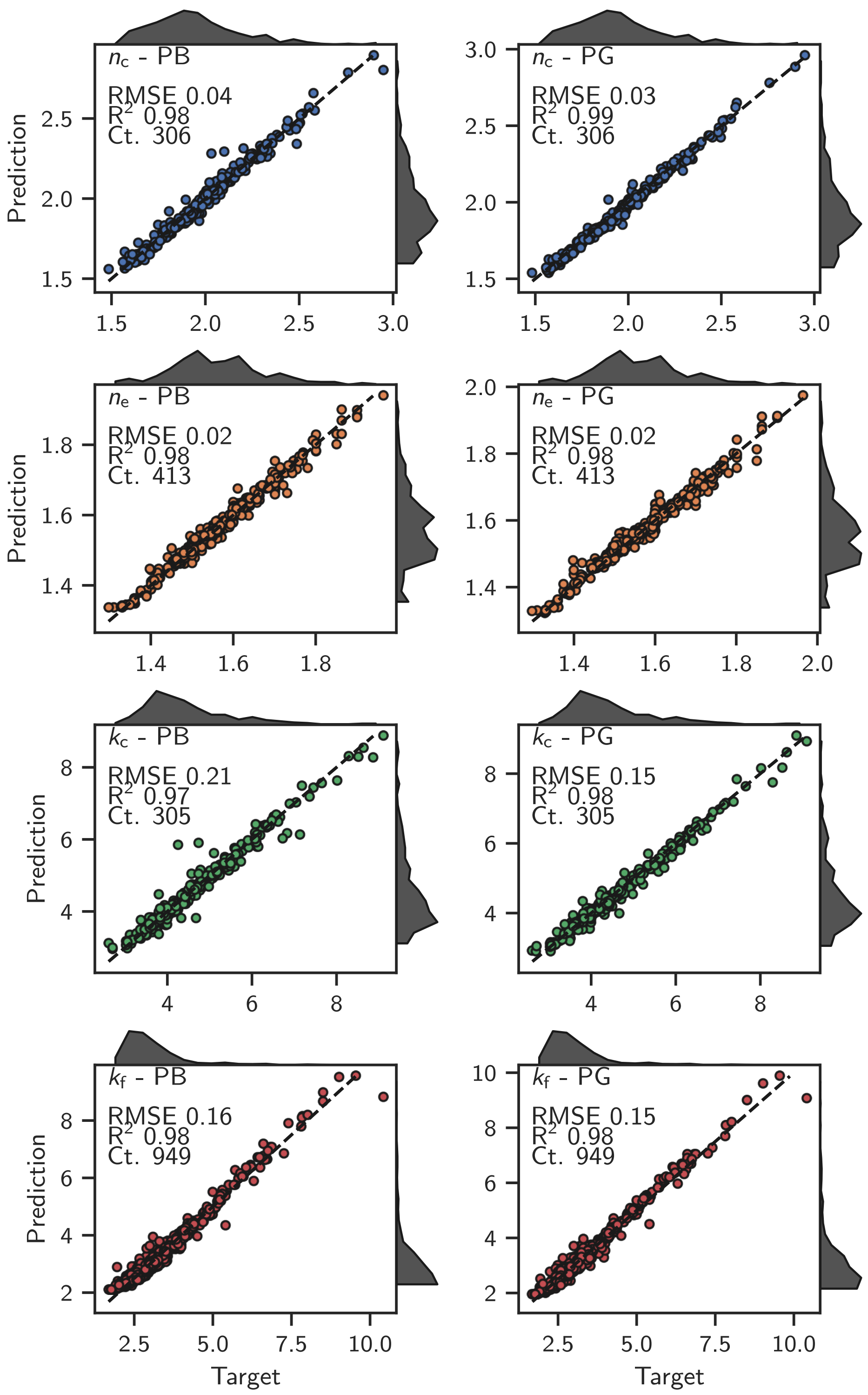

Supplementary Figure S12: Meta learner parity plots of the optical & dielectric properties' test data sets for polyBERT (PB) and Polymer Genome (PG) fingerprints. Data point frequencies of homopolymers are indicated in black in the margins of the plots. RMSE, R^2, and Ct. denote the root-mean-square error, coefficient of determination, and number of data points. n_c, n_e, k_c, and k_f stand for refractive index (DFT), refractive index (exp.), dielectric constant (DFT), and dielectric constant at freq. $\mathbf{f}$.

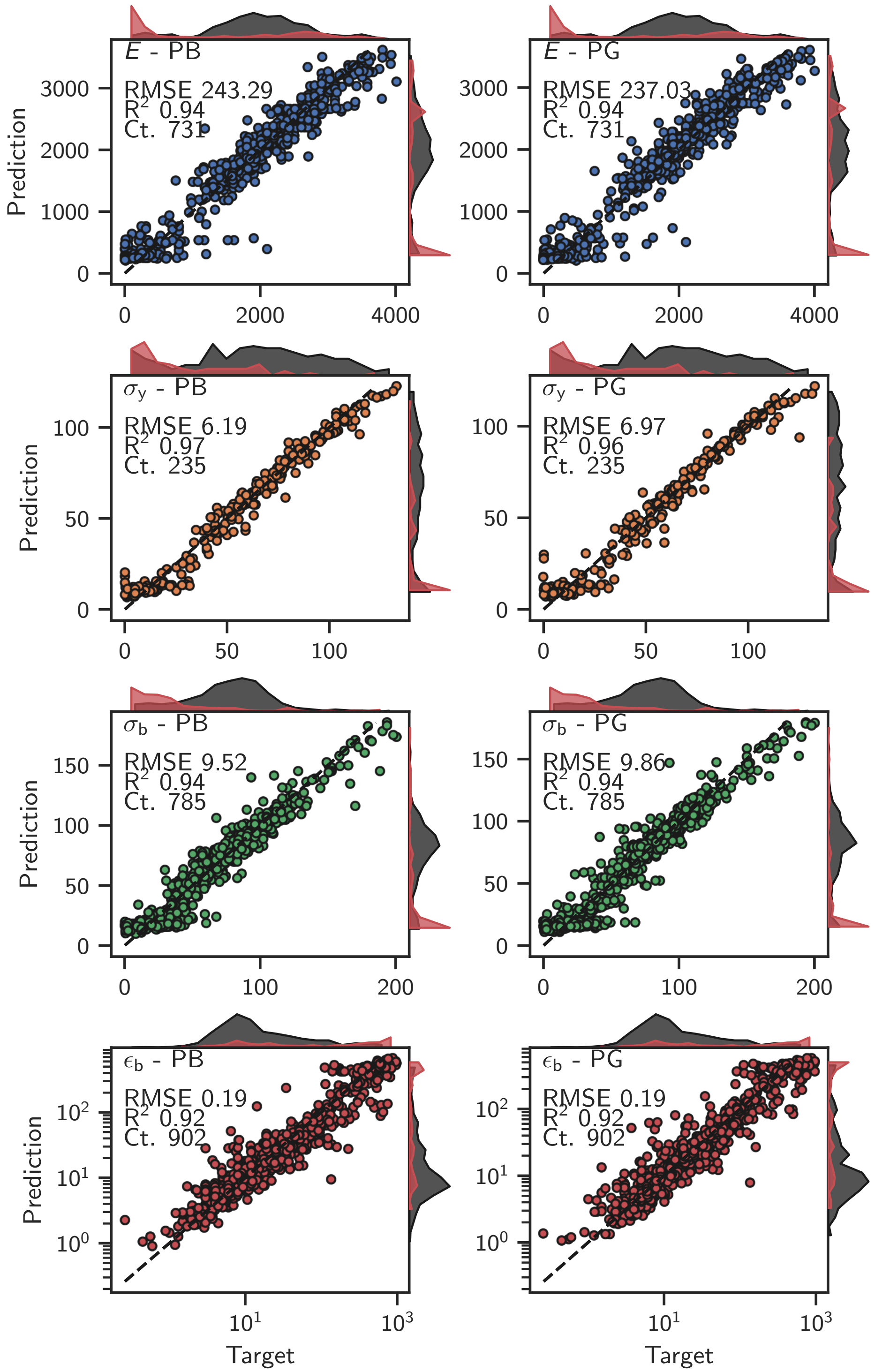

Supplementary Figure S13: Meta learner parity plots of the mechanical properties' test data sets for polyBERT (PB) and Polymer Genome (PG) fingerprints. Data point frequencies of homopolymers and copolymers are indicated in black and red in the margins of the plots. RMSE, R^2, and Ct. denote the root-mean-square error, coefficient of determination, and number of data points. E, σ_y, σ_b, and ϵ_b stand for young's modulus, tensile strength at yield, tensile strength at break, and elongation at break.

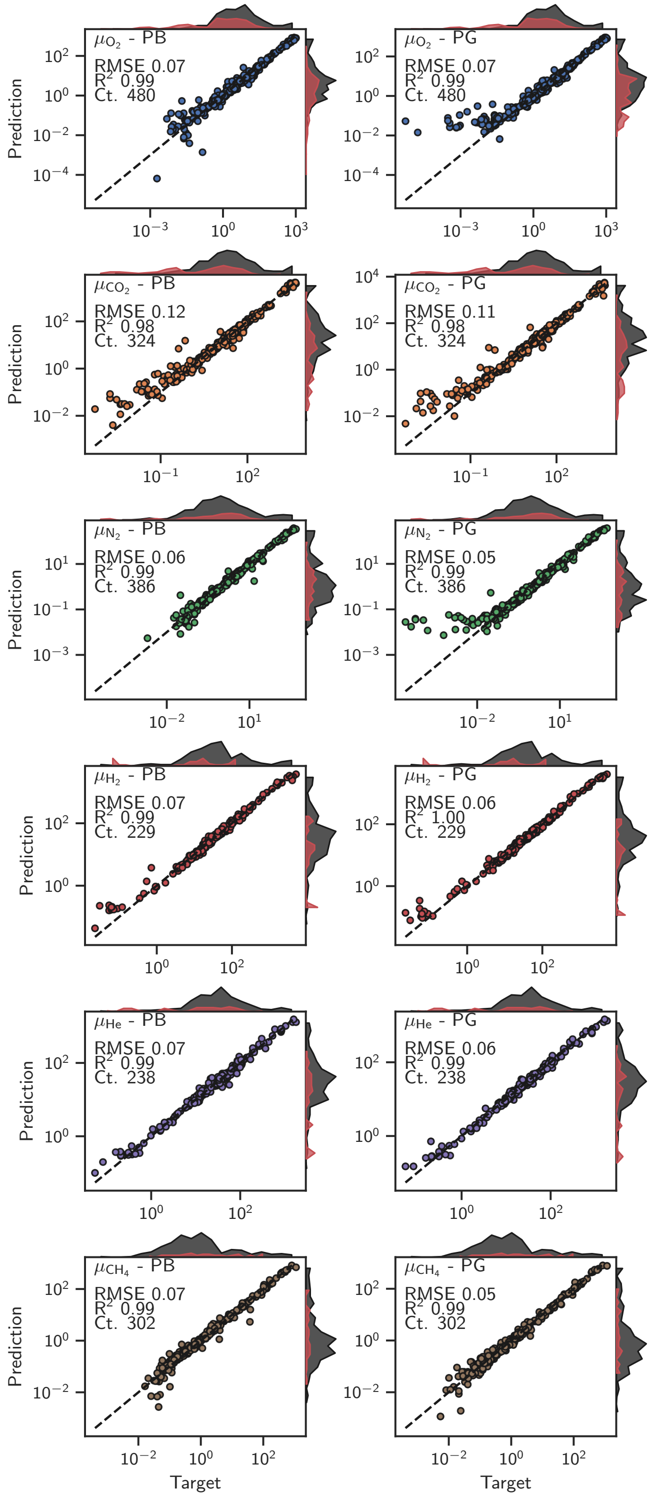

Supplementary Figure S14: Meta learner parity plots of the permeability properties' test data sets for polyBERT (PB) and Polymer Genome (PG) fingerprints. Data point frequencies of homopolymers and copolymers are indicated in black and red in the margins of the plots. RMSE, R^2, and Ct. denote the root-mean-square error, coefficient of determination, and number of data points. μ_{O_2}, μ_{CO_2}, μ_{N_2}, μ_{H_2}, μ_{He}, and μ_{CH_4} stand for O_2 gas permeability, CO_2 gas permeability, N_2 gas permeability, H_2 gas permeability, He gas permeability, and CH_4 gas permeability.

Supplementary Table S1: The root-mean-square error (RMSE) and coefficient of determination (R^2) averages of the five cross-validation test data sets along with the standard deviations (1σ), and meta learner RMSE and R^2 values for polyBERT (PB) and Polymer Genome (PG) fingerprints. Values are averages over the polymers in the data set. The best method of the two is highlighted for each metric and property in bold font. The six gas permeabilities (μ_x) and elongation at break (ϵ_b) are trained on log base 10 scale ($x \mapsto \log_{10}(x+1)$) and reported on this scale. T_g, T_m, and T_d stand for glass transition, melting, and degradation temperature. c_p, E_{at}, O_i, X_c, X_e, and ρ stand for heat capacity, atomization energy, limiting oxygen index, crystallization tendency (DFT), crystallization tendency (exp.), and density. E_{gc}, E_{gb}, E_{ea}, E_i, E_{ib}, and δ stand for band gap (chain), band gap (bulk), electron affinity, ionization energy, electronic injection barrier, and cohesive energy density. n_c, n_e, k_c, and k_f stand for refractive index (DFT), refractive index (exp.), dielectric constant (DFT), and dielectric constant at freq. **f**. E, σ_y, σ_b, and ϵ_b stand for young's modulus, tensile strength at yield, tensile strength at break, and elongation at break. μ_{O_2}, μ_{CO_2}, μ_{N_2}, μ_{H_2}, μ_{He}, and μ_{CH_4} stand for O_2, CO_2, N_2, H_2, He, and CH_4 gas permeability.

	Cross-validation				Meta			
	RMSE		R2		RMSE		R2	
	PB	PG	PB	PG	PB	PG	PB	PG
T_g	30.18±0.88	**28.78±0.40**	0.92±0.01	**0.92±0.00**	**12.66**	12.80	**0.99**	0.98
T_m	40.21±1.32	**39.87±1.72**	0.84±0.02	**0.84±0.02**	**16.83**	17.15	**0.97**	0.97
T_d	62.76±2.78	**61.08±1.63**	0.70±0.03	**0.72±0.02**	**23.30**	23.42	**0.96**	0.96
c_p	0.23±0.04	**0.18±0.04**	0.61±0.11	**0.76±0.10**	0.10	**0.07**	0.93	**0.97**
E_{at}	0.13±0.01	**0.09±0.01**	0.85±0.02	**0.94±0.02**	0.06	**0.04**	0.97	**0.98**
O_i	8.11±1.37	**7.72±1.35**	0.57±0.16	**0.61±0.16**	3.65	**2.76**	0.92	**0.96**
X_c	**16.68±2.55**	17.13±1.11	**0.50±0.11**	0.47±0.06	**7.54**	7.61	**0.90**	0.90
X_e	13.50±4.00	**13.41±2.61**	**0.44±0.28**	0.41±0.32	6.77	**6.07**	0.87	**0.90**
ρ	0.10±0.01	**0.09±0.01**	0.75±0.03	**0.81±0.05**	0.05	**0.04**	0.95	**0.96**
E_{gc}	0.48±0.04	**0.48±0.05**	0.89±0.02	**0.90±0.02**	**0.20**	0.23	**0.98**	0.98
E_{gb}	0.49±0.04	**0.48±0.04**	0.93±0.01	**0.94±0.01**	0.26	**0.24**	0.98	**0.98**
E_{ea}	**0.28±0.04**	0.28±0.04	**0.93±0.03**	0.93±0.03	0.13	**0.12**	0.99	**0.99**
E_i	0.42±0.06	**0.39±0.03**	0.82±0.07	**0.85±0.03**	0.19	**0.16**	0.96	**0.97**
E_{ib}	0.27±0.03	**0.27±0.03**	0.71±0.05	**0.72±0.06**	**0.13**	0.13	**0.94**	0.93
δ	28.43±9.44	**25.48±7.71**	0.57±0.36	**0.65±0.28**	**9.40**	10.52	**0.96**	0.95
n_c	0.09±0.03	**0.08±0.03**	0.86±0.06	**0.89±0.05**	0.04	**0.03**	0.98	**0.99**
n_e	0.05±0.01	**0.05±0.01**	0.76±0.06	**0.78±0.02**	**0.02**	0.02	**0.98**	0.98
k_c	**0.42±0.11**	0.44±0.07	**0.86±0.06**	0.84±0.04	0.21	**0.15**	0.97	**0.98**
k_f	0.31±0.05	**0.29±0.04**	0.91±0.03	**0.92±0.02**	0.16	**0.15**	0.98	**0.98**
E	495.94±71.18	**494.54±49.88**	0.75±0.07	**0.75±0.04**	243.29	**237.03**	0.94	**0.94**
σ_y	14.96±2.97	**14.35±2.58**	0.80±0.08	**0.81±0.08**	**6.19**	6.97	**0.97**	0.96
σ_b	19.20±1.64	**18.13±2.52**	0.76±0.05	**0.79±0.04**	**9.52**	9.86	**0.94**	0.94
ϵ_b	**0.41±0.01**	0.41±0.01	**0.62±0.06**	0.61±0.07	0.19	**0.19**	0.92	**0.92**
μ_{O_2}	0.13±0.02	**0.13±0.02**	0.96±0.01	**0.96±0.01**	0.07	**0.07**	0.99	**0.99**
μ_{CO_2}	0.22±0.03	**0.22±0.05**	**0.94±0.02**	0.94±0.03	0.12	**0.11**	0.98	**0.98**
μ_{N_2}	0.12±0.04	**0.12±0.03**	0.96±0.03	**0.96±0.03**	0.06	**0.05**	0.99	**0.99**
μ_{H_2}	0.14±0.03	**0.13±0.03**	0.97±0.01	**0.97±0.01**	0.07	**0.06**	0.99	**1.00**
μ_{He}	0.14±0.03	**0.13±0.02**	0.95±0.02	**0.96±0.02**	0.07	**0.06**	0.99	**0.99**
μ_{CH_4}	0.14±0.04	**0.13±0.04**	0.95±0.03	**0.96±0.03**	0.07	**0.05**	0.99	**0.99**

Supplementary Table S2: The homopolymer root-mean-square error (RMSE) and coefficient of determination (R^2) averages of the five cross-validation test data sets along with the standard deviations (1σ), and meta learner RMSE and R^2 values for polyBERT (PB) and Polymer Genome (PG) fingerprints. Values are averages over the homopolymers in the data set. The best method of the two is highlighted for each metric and property in bold font. The six gas permeabilities (μ_x) and elongation at break (ϵ_b) are trained on log base 10 scale ($x \mapsto \log_{10}(x+1)$) and reported on this scale. T_g, T_m, and T_d stand for glass transition, melting, and degradation temperature. c_p, E_{at}, O_i, X_c, X_e, and ρ stand for heat capacity, atomization energy, limiting oxygen index, crystallization tendency (DFT), crystallization tendency (exp.), and density. E_{gc}, E_{gb}, E_{ea}, E_i, E_{ib}, and δ stand for band gap (chain), band gap (bulk), electron affinity, ionization energy, electronic injection barrier, and cohesive energy density. n_c, n_e, k_c, and k_f stand for refractive index (DFT), refractive index (exp.), dielectric constant (DFT), and dielectric constant at freq. **f**. E, σ_y, σ_b, and ϵ_b stand for young's modulus, tensile strength at yield, tensile strength at break, and elongation at break. μ_{O_2}, μ_{CO_2}, μ_{N_2}, μ_{H_2}, μ_{He}, and μ_{CH_4} stand for O_2, CO_2, N_2, H_2, He, and CH_4 gas permeability.

	Cross-validation				Meta			
	RMSE		R2		RMSE		R2	
	PB	PG	PB	PG	PB	PG	PB	PG
T_g	35.70±0.55	**34.20±0.60**	0.89±0.00	**0.90±0.00**	**12.83**	13.63	**0.99**	0.98
T_m	48.25±2.29	**48.01±2.19**	0.80±0.02	**0.80±0.02**	**15.50**	16.36	**0.98**	0.98
T_d	65.16±3.98	**63.17±2.49**	0.68±0.05	**0.70±0.02**	**20.49**	20.76	**0.97**	0.97
c_p	0.23±0.04	**0.18±0.04**	0.61±0.11	**0.76±0.10**	0.10	**0.07**	0.93	**0.97**
E_{at}	0.13±0.01	**0.09±0.01**	0.85±0.02	**0.94±0.02**	0.06	**0.04**	0.97	**0.98**
O_i	8.11±1.37	**7.72±1.35**	0.57±0.16	**0.61±0.16**	3.65	**2.76**	0.92	**0.96**
X_c	**16.68±2.55**	17.13±1.11	**0.50±0.11**	0.47±0.06	**7.54**	7.61	**0.90**	0.90
X_e	13.50±4.00	**13.41±2.61**	**0.44±0.28**	0.41±0.32	6.77	**6.07**	0.87	**0.90**
ρ	0.10±0.01	**0.09±0.01**	0.75±0.03	**0.81±0.05**	0.05	**0.04**	0.95	**0.96**
E_{gc}	0.48±0.04	**0.48±0.05**	0.89±0.02	**0.90±0.02**	**0.20**	0.23	**0.98**	0.98
E_{gb}	0.49±0.04	**0.48±0.04**	0.93±0.01	**0.94±0.01**	0.26	**0.24**	0.98	**0.98**
E_{ea}	**0.28±0.04**	0.28±0.04	**0.93±0.03**	0.93±0.03	0.13	**0.12**	0.99	**0.99**
E_i	0.42±0.06	**0.39±0.03**	0.82±0.07	**0.85±0.03**	0.19	**0.16**	0.96	**0.97**
E_{ib}	0.27±0.03	**0.27±0.03**	0.71±0.05	**0.72±0.06**	**0.13**	0.13	**0.94**	0.93
δ	28.43±9.44	**25.48±7.71**	0.57±0.36	**0.65±0.28**	**9.40**	10.52	**0.96**	0.95
n_c	0.09±0.03	**0.08±0.03**	0.86±0.06	**0.89±0.05**	0.04	**0.03**	0.98	**0.99**
n_e	0.05±0.01	**0.05±0.01**	0.76±0.06	**0.78±0.02**	**0.02**	0.02	**0.98**	0.98
k_c	**0.42±0.11**	0.44±0.07	**0.86±0.06**	0.84±0.04	0.21	**0.15**	0.97	**0.98**
k_f	0.31±0.05	**0.29±0.04**	0.91±0.03	**0.92±0.02**	0.16	**0.15**	0.98	**0.98**
E	523.50±70.66	**506.06±68.04**	0.52±0.13	**0.56±0.11**	216.87	**213.03**	0.92	**0.92**
σ_y	14.96±3.35	**13.82±2.82**	0.77±0.07	**0.81±0.04**	**5.88**	6.49	**0.97**	0.96
σ_b	19.95±1.90	**18.40±2.80**	0.60±0.09	**0.66±0.06**	**8.40**	8.50	**0.93**	0.93
ϵ_b	**0.40±0.02**	0.41±0.02	**0.45±0.11**	0.42±0.14	**0.16**	0.16	**0.91**	0.91
μ_{O_2}	0.12±0.03	**0.11±0.03**	0.97±0.01	**0.98±0.01**	0.06	**0.05**	0.99	**0.99**
μ_{CO_2}	**0.20±0.05**	0.20±0.07	**0.95±0.02**	0.95±0.03	**0.10**	0.11	**0.99**	0.98
μ_{N_2}	0.10±0.02	**0.09±0.03**	0.97±0.01	**0.98±0.01**	0.04	**0.04**	1.00	**1.00**
μ_{H_2}	0.14±0.03	**0.13±0.03**	0.96±0.02	**0.97±0.01**	0.07	**0.06**	0.99	**0.99**
μ_{He}	0.14±0.03	**0.13±0.02**	0.94±0.04	**0.95±0.03**	0.06	**0.05**	0.99	**0.99**
μ_{CH_4}	0.10±0.01	**0.09±0.02**	0.98±0.01	**0.98±0.01**	**0.04**	0.04	**1.00**	1.00

Supplementary Table S3: The copolymer root-mean-square error (RMSE) and coefficient of determination (R^2) averages of the five cross-validation test data sets along with the standard deviations (1σ), and meta learner RMSE and R^2 values for polyBERT (PB) and Polymer Genome (PG) fingerprints. Values are averages over the copolymers in the data set. The best method of the two is highlighted for each metric and property in bold font. The six gas permeabilities (μ_x) and elongation at break (ϵ_b) are trained on log base 10 scale ($x \mapsto \log_{10}(x+1)$) and reported on this scale. T_g, T_m, and T_d stand for glass transition, melting, and degradation temperature. c_p, E_at, O_i, X_c, X_e, and ρ stand for heat capacity, atomization energy, limiting oxygen index, crystallization tendency (DFT), crystallization tendency (exp.), and density. E_gc, E_gb, E_ea, E_i, E_ib, and δ stand for band gap (chain), band gap (bulk), electron affinity, ionization energy, electronic injection barrier, and cohesive energy density. n_c, n_e, k_c, and k_f stand for refractive index (DFT), refractive index (exp.), dielectric constant (DFT), and dielectric constant at freq. **f**. E, σ_y, σ_b, and ϵ_b stand for young's modulus, tensile strength at yield, tensile strength at break, and elongation at break. $\mu_{\mathrm{O_2}}$, $\mu_{\mathrm{CO_2}}$, $\mu_{\mathrm{N_2}}$, $\mu_{\mathrm{H_2}}$, μ_{He}, and $\mu_{\mathrm{CH_4}}$ stand for O_2, CO_2, N_2, H_2, He, and CH_4 gas permeability.

	Cross-validation				Meta			
	RMSE		R2		RMSE		R2	
	PB	PG	PB	PG	PB	PG	PB	PG
T_g	18.36±2.57	**17.14±0.61**	0.96±0.01	**0.96±0.00**	12.39	**11.37**	0.98	**0.98**
T_m	25.27±1.25	**24.53±1.73**	0.89±0.01	**0.90±0.02**	18.51	**18.18**	0.94	**0.94**
T_d	54.02±3.75	**53.30±4.96**	0.75±0.04	**0.75±0.04**	30.91	**30.71**	0.92	**0.92**
E	**441.56±95.35**	475.90±62.86	**0.86±0.05**	0.83±0.04	286.65	**276.76**	0.95	**0.95**
σ_y	12.28±5.99	**12.27±6.82**	**0.65±0.14**	0.64±0.20	**6.97**	8.14	**0.93**	0.91
σ_b	**17.48±2.18**	17.55±3.26	**0.83±0.03**	0.82±0.05	**11.58**	12.27	**0.93**	0.92
ϵ_b	0.43±0.07	**0.41±0.08**	0.71±0.09	**0.73±0.09**	0.27	**0.26**	0.89	**0.90**
$\mu_{\mathrm{O_2}}$	**0.15±0.05**	0.15±0.05	**0.89±0.05**	0.88±0.06	**0.09**	0.10	**0.96**	0.96
$\mu_{\mathrm{CO_2}}$	0.23±0.07	**0.22±0.09**	0.88±0.05	**0.89±0.06**	0.16	**0.12**	0.95	**0.97**
$\mu_{\mathrm{N_2}}$	**0.14±0.10**	0.16±0.09	**0.87±0.12**	0.84±0.10	0.11	**0.07**	0.93	**0.97**
$\mu_{\mathrm{H_2}}$	0.11±0.06	**0.10±0.05**	0.97±0.03	**0.97±0.02**	0.06	**0.05**	0.99	**0.99**
μ_{He}	0.14±0.05	**0.12±0.05**	0.91±0.12	**0.94±0.05**	0.09	**0.07**	0.98	**0.99**
$\mu_{\mathrm{CH_4}}$	0.30±0.15	**0.25±0.14**	0.60±0.32	**0.72±0.27**	0.16	**0.08**	0.93	**0.98**

Supplementary Table S4: Key figures of the polymer fingerprints. The sparsity is computed from the ratio of zeros and non-zeros of all data points.

	Dimensions	Sparsity [%]	Min.	Max.	Device
polyBERT	600	0	-17.8	30.1	CPU & GPU
Polymer Genome	945	93.9	0	12.2	CPU

Supplementary Table S5: Minimum, mean, and maximum of polyBERT-based property predictions for the data set of 100 million hypothetical polymers. T_g, T_m, and T_d stand for glass transition, melting, and degradation temperature. c_p, E_{at}, O_i, X_c, X_e, and ρ stand for heat capacity, atomization energy, limiting oxygen index, crystallization tendency (DFT), crystallization tendency (exp.), and density. E_{gc}, E_{gb}, E_{ea}, E_i, E_{ib}, and δ stand for band gap (chain), band gap (bulk), electron affinity, ionization energy, electronic injection barrier, and cohesive energy density. n_c, n_e, k_c, and k_f stand for refractive index (DFT), refractive index (exp.), dielectric constant (DFT), and dielectric constant at freq. **f**. E, σ_y, σ_b, and ϵ_b stand for young's modulus, tensile strength at yield, tensile strength at break, and elongation at break. μ_{O_2}, μ_{CO_2}, μ_{N_2}, μ_{H_2}, μ_{He}, and μ_{CH_4} stand for O_2, CO_2, N_2, H_2, He, and CH_4 gas permeability.

	Unit	Min	Mean	Max
T_g	K	162.21	498.71	836.81
T_m	K	275.11	592.19	873.36
T_d	K	394.11	713.01	1185.81
c_p	$Jg^{-1}K^{-1}$	0.87	1.28	2.33
E_{at}	eV $atom^{-1}$	-6.69	-6.03	-4.93
O_i	%	15.11	32.47	72.87
X_c	%	3.90	41.35	100.50
X_e	%	5.19	38.25	92.05
ρ	g cm^{-3}	0.88	1.30	2.07
E_{gc}	eV	0.42	3.42	10.39
E_{gb}	eV	0.78	3.06	10.59
E_{ea}	eV	0.59	2.26	5.12
E_i	eV	3.81	5.82	10.63
E_{ib}	eV	2.00	3.34	5.42
δ	cal cm^{-3}	32.15	132.43	293.57
n_c		1.55	1.95	3.22
n_e		1.33	1.63	2.21
k_c		2.92	4.45	10.46
k_f		2.10	3.45	9.86
E	MPa	177.56	2016.50	3884.81
σ_y	MPa	5.94	67.52	123.94
σ_b	MPa	9.05	83.44	184.27
ϵ_b		0.85	14.79	565.19
μ_{O_2}	barrer	-0.36	5.97	3047.37
μ_{CO_2}	barrer	-0.40	25.41	21933.14
μ_{N_2}	barrer	-0.39	1.57	892.52
μ_{H_2}	barrer	-0.41	41.68	14659.80
μ_{He}	barrer	-0.34	34.99	6484.88
μ_{CH_4}	barrer	-0.47	1.74	1683.41

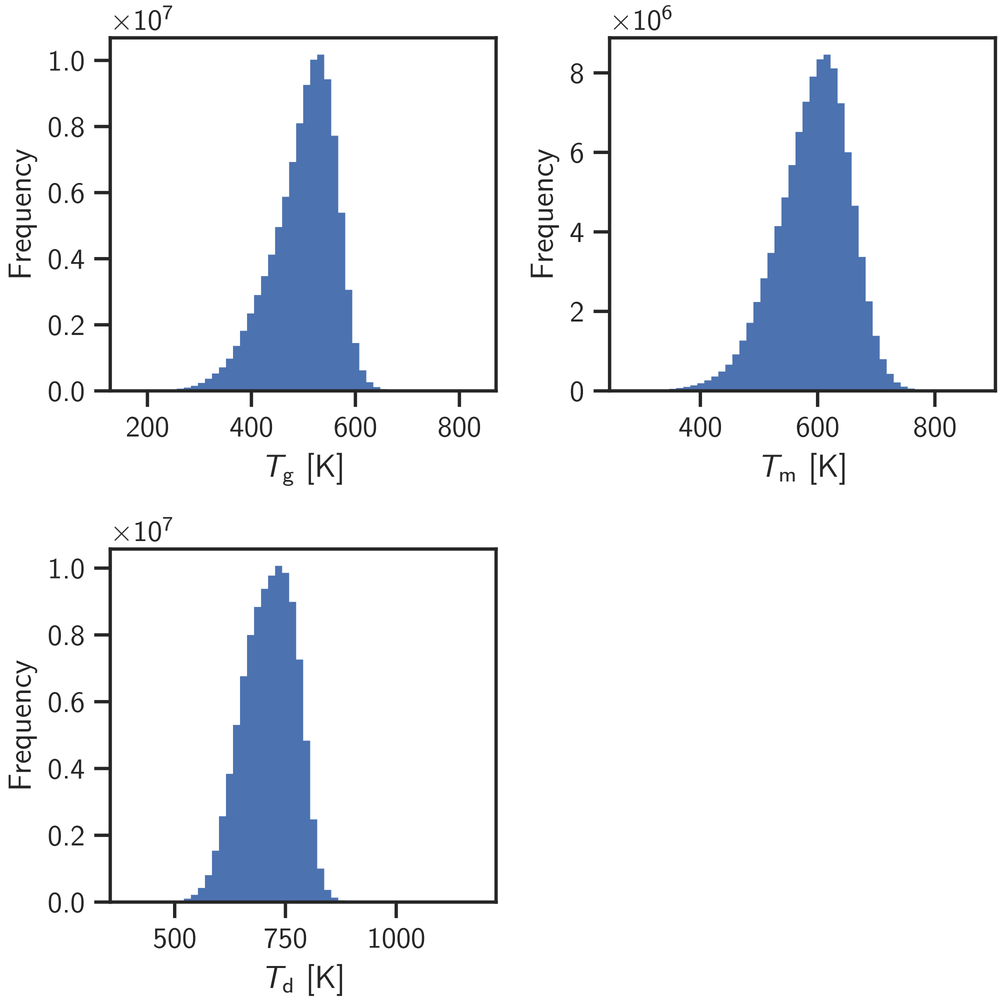

Supplementary Figure S15: Histograms of thermal properties for 100 million hypothetical polymers. T_g, T_m, and T_d stand for glass transition temperature, melting temperature, and degradation temperature.

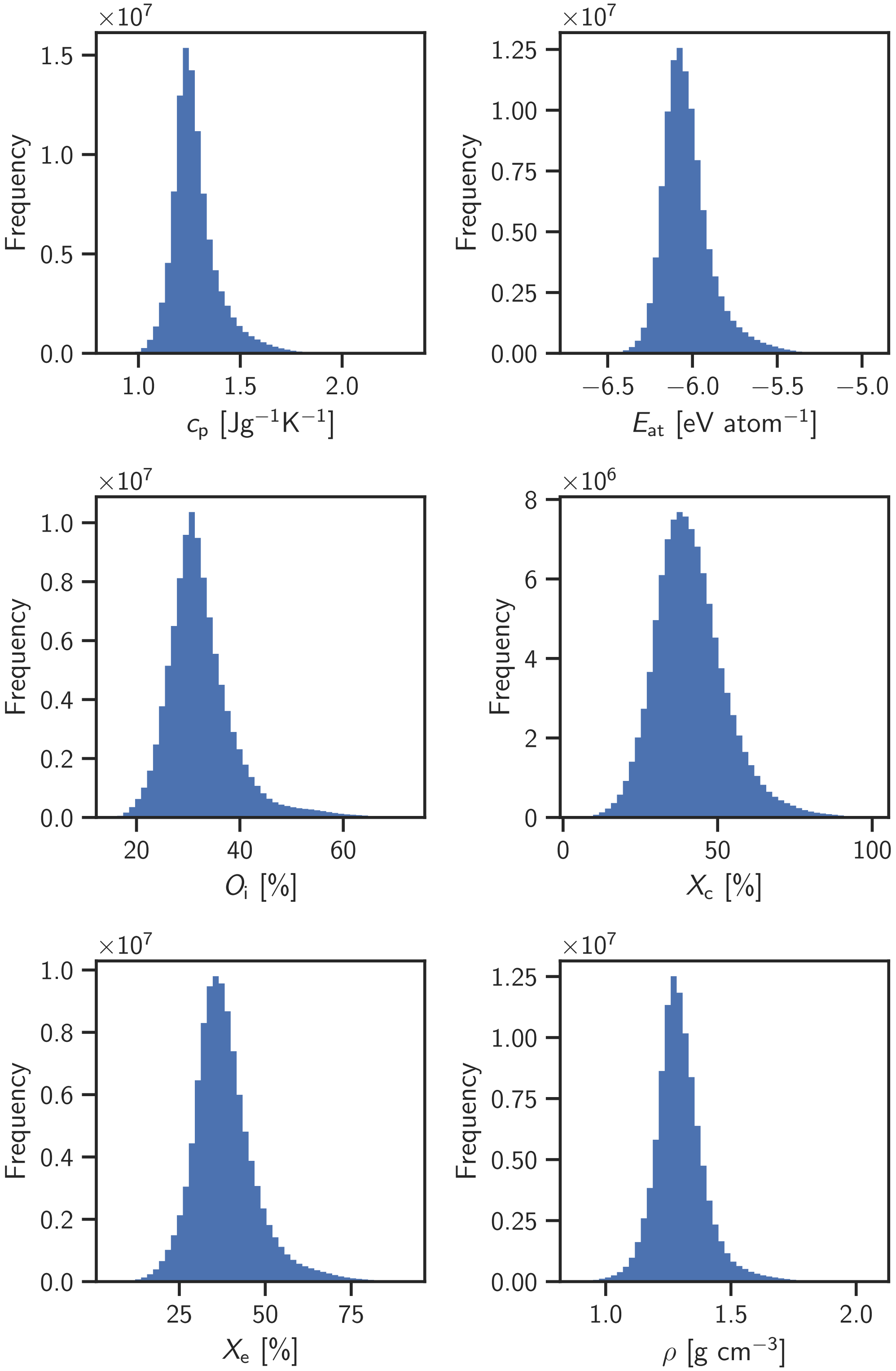

Supplementary Figure S16: Histograms of thermodynamic & physical properties for 100 million hypothetical polymers. c_{p}, E_{at}, O_{i}, X_{c}, X_{e}, and ρ stand for heat capacity, atomization energy, limiting oxygen index, crystallization tendency (DFT), crystallization tendency (exp.), and density.

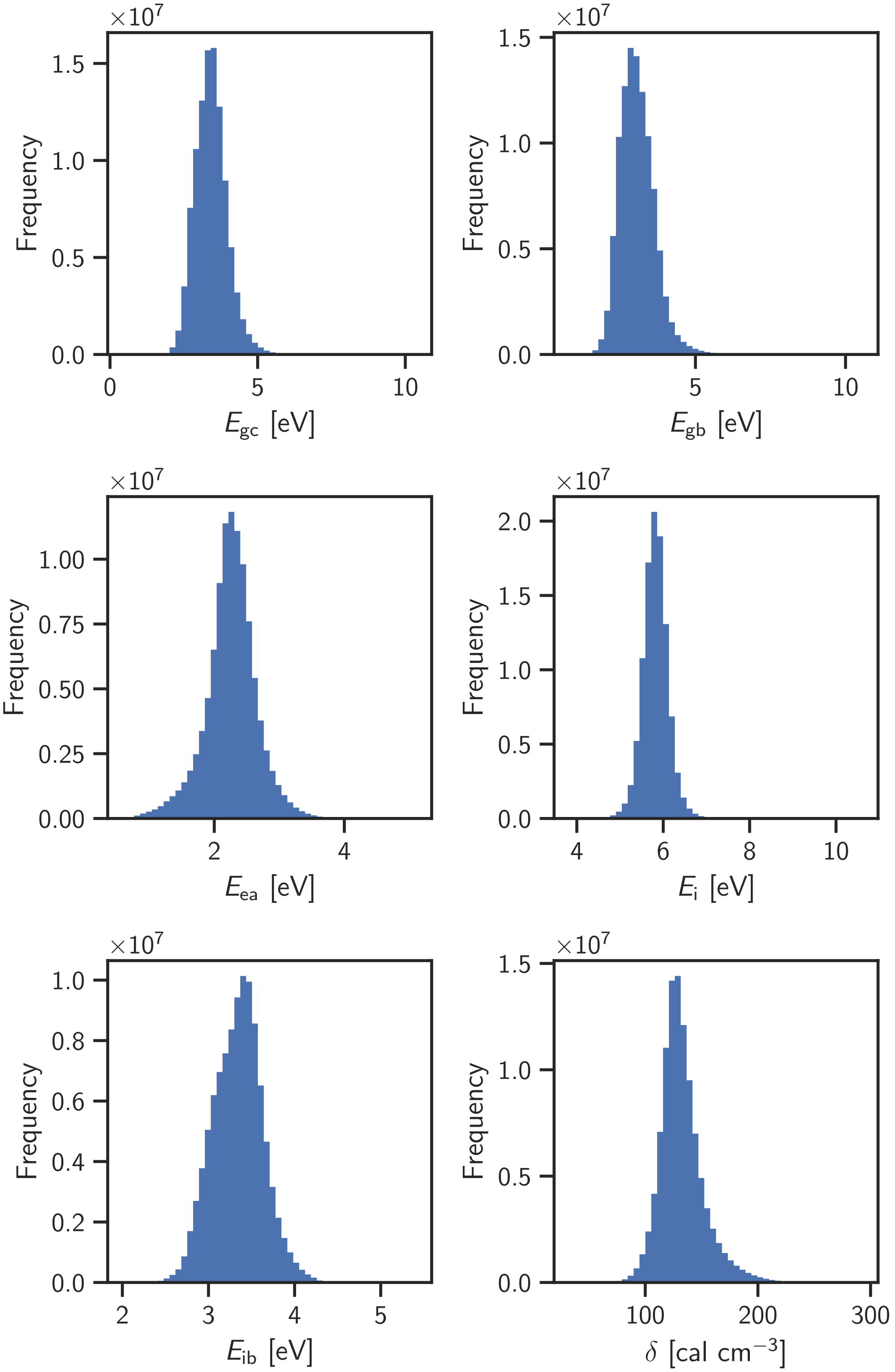

Supplementary Figure S17: Histograms of electronic properties for 100 million hypothetical polymers. E_{gc}, E_{gb}, E_{ea}, E_{i}, E_{ib}, and δ stand for band gap (chain), band gap (bulk), electron affinity, ionization energy, electronic injection barrier, and cohesive energy density.

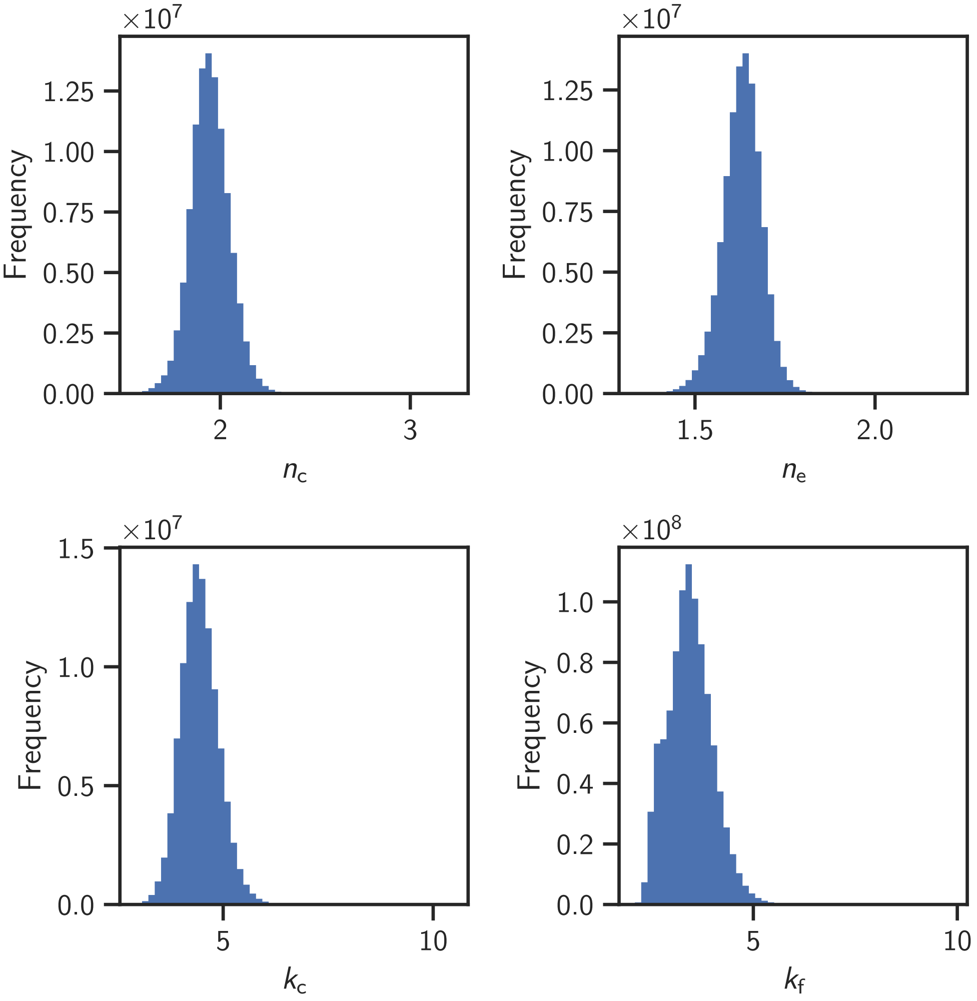

Supplementary Figure S18: Histograms of optical & dielectric properties for 100 million hypothetical polymers. n_c, n_e, k_c, and k_f stand for refractive index (DFT), refractive index (exp.), dielectric constant (DFT), and dielectric constant at freq. **f**.

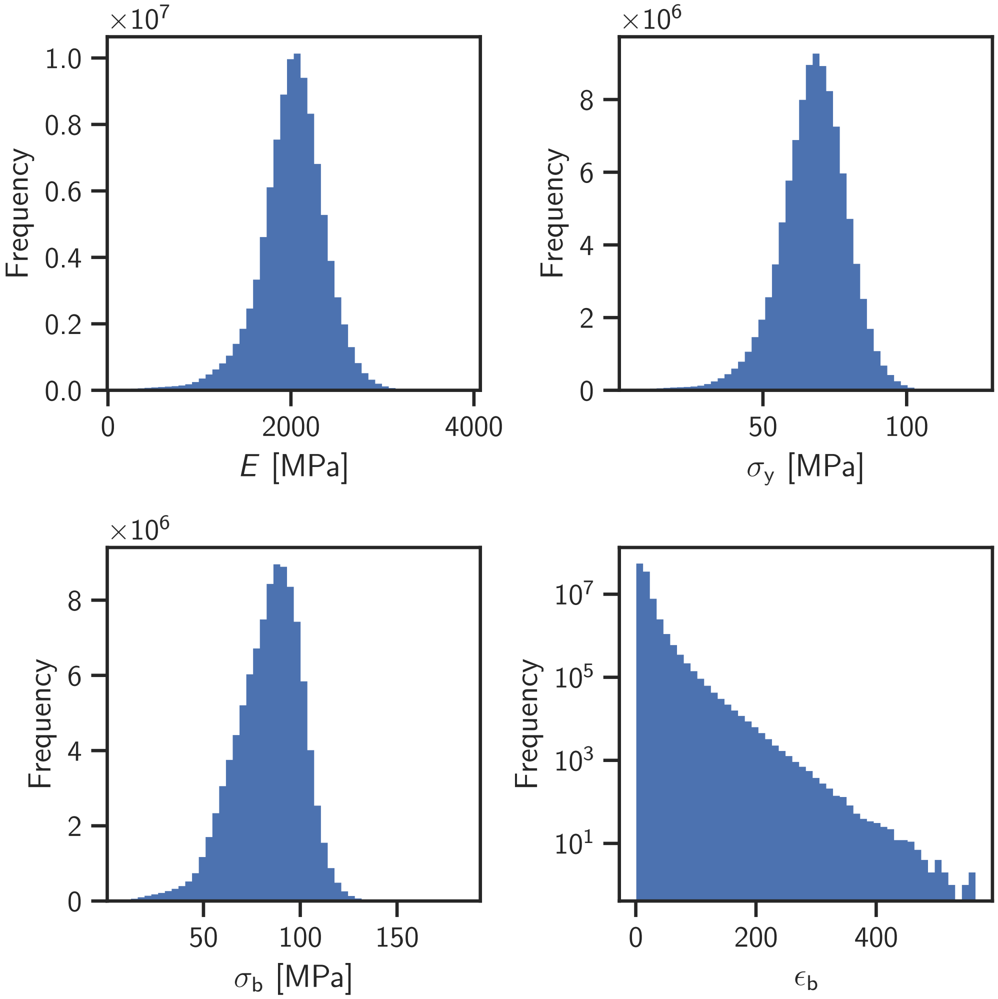

Supplementary Figure S19: Histograms of mechanical properties for 100 million hypothetical polymers. E, σ_y, σ_b, and ϵ_b stand for young's modulus, tensile strength at yield, tensile strength at break, and elongation at break.

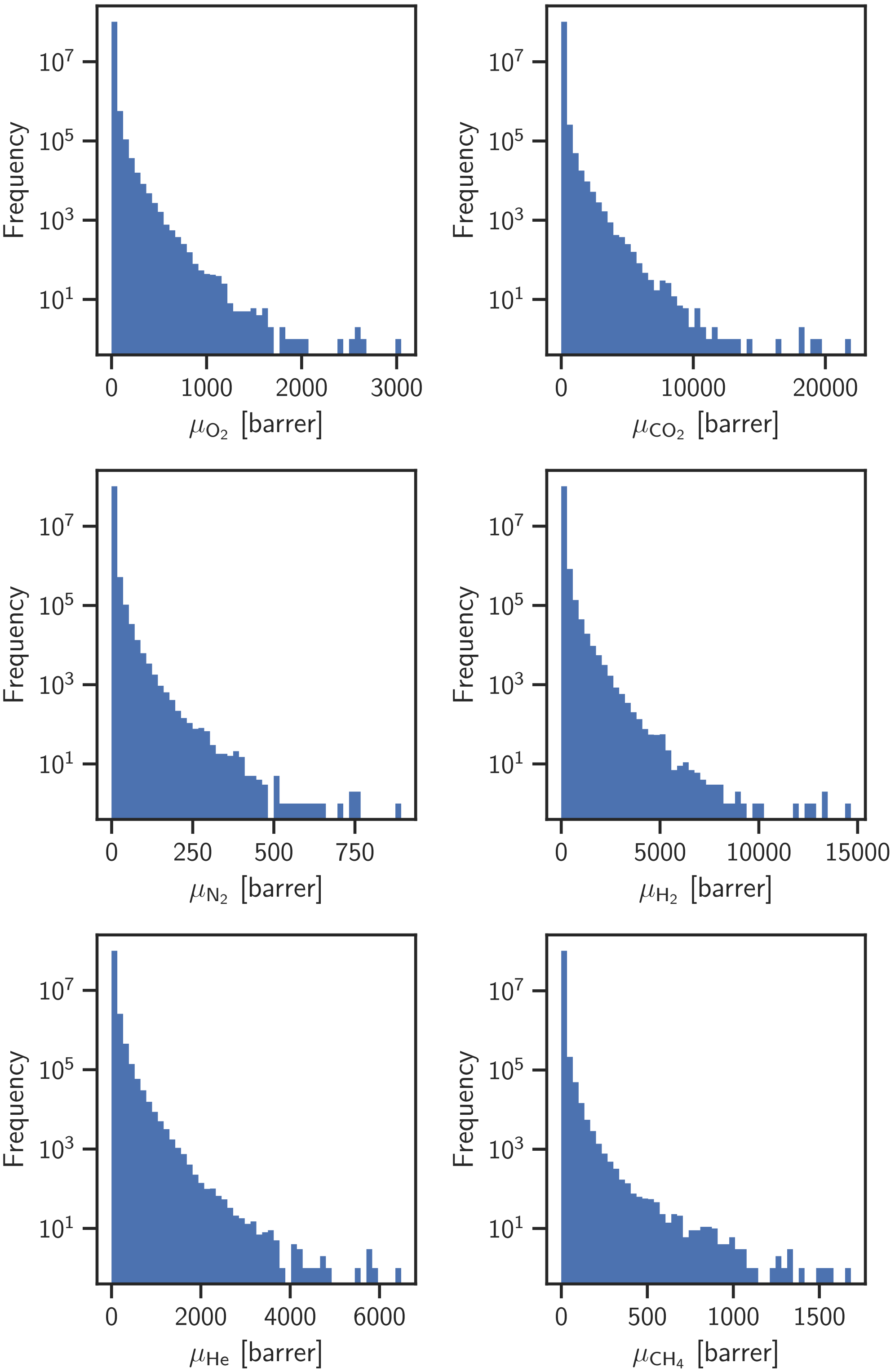

Supplementary Figure S20: Histograms of permeability properties for 100 million hypothetical polymers. μ_{O_2}, μ_{CO_2}, μ_{N_2}, μ_{H_2}, μ_{He}, and μ_{CH_4} stand for O_2 gas permeability, CO_2 gas permeability, N_2 gas permeability, H_2 gas permeability, He gas permeability, and CH_4 gas permeability.

Supplementary References

(1) Devlin, J.; Chang, M.-W.; Lee, K.; Toutanova, K. BERT: Pre-training of Deep Bidirectional Transformers for Language Understanding. *arXiv* **2018**, DOI: 10.48550/arXiv.1810.04805.

(2) Liu, Y.; Ott, M.; Goyal, N.; Du, J.; Joshi, M.; Chen, D.; Levy, O.; Lewis, M.; Zettlemoyer, L.; Stoyanov, V. RoBERTa: A Robustly Optimized BERT Pretraining Approach. *arXiv* **2019**, DOI: 10.48550/arXiv.1907.11692.

(3) Sanh, V.; Debut, L.; Chaumond, J.; Wolf, T. DistilBERT, a distilled version of BERT: smaller, faster, cheaper and lighter. *arXiv* **2019**, DOI: 10.48550/arXiv.1910.01108.

(4) He, P.; Liu, X.; Gao, J.; Chen, W. DeBERTa: Decoding-enhanced BERT with Disentangled Attention. *arXiv* **2020**, DOI: 10.48550/arXiv.2006.03654.
